# Genotype-Specific Incidence and Clearance of Human Papillomavirus in Oral Mucosa of Women: A Six-Year Follow-Up Study

**DOI:** 10.1371/journal.pone.0053413

**Published:** 2013-01-03

**Authors:** Karolina Louvanto, Jaana Rautava, Jaana Willberg, Lilli Wideman, Kari Syrjänen, Seija Grénman, Stina Syrjänen

**Affiliations:** 1 Department of Oral Pathology, Institute of Dentistry, Faculty of Medicine and Medicity Research Laboratory, University of Turku, Turku, Finland; 2 Department of Obstetrics and Gynecology, Turku University Hospital, University of Turku, Turku, Finland; 3 Department of Oncology and Radiotherapy, Turku University Hospital, Turku, Finland; 4 Teaching and Research Institute, Barretos Cancer Hospital, Barretos, São Paulo, Brazil; Blood Systems Research Institute, United States of America

## Abstract

**Background:**

There are no previous longitudinal studies on genotype-specific natural history of human papillomavirus (HPV) infections in oral mucosa of women.

**Methods:**

In the Finnish Family HPV Study, 329 pregnant women were enrolled and followed up. HPV-genotyping of oral scrapings was performed with nested PCR and Multimetrix® test (Progen, Heidelberg, Germany). Incidence and clearance times and rates for each HPV-genotype identified in oral mucosa were determined. Predictors for incident and cleared HPV infections for species 7/9 genotypes were analyzed using Poisson regression model.

**Results:**

Altogether, 115 baseline HPV-negative women acquired incident oral HPV infection, and 79 women cleared their infection. HPV16 and multiple HPVs most frequently caused incident infections (65% and 12%) in 13.3 and 17.1 months respectively, followed by HPV58, HPV18 and HPV6 (close to 5% each) in 11–24 months. HPV58, HPV18 and HPV66 were the most common to clear. HPV6 and HPV11 had the shortest clearance times, 4.6 months and 2.5 months, and the highest clearance rates, 225.5/1000 wmr and 400/1000 wmr, respectively. The protective factors for incident oral HPV-species 7/9 infections were 1) new pregnancy during follow-up and 2) having the same sexual partner during FU. Increased clearance was related with older age and a history of atopic reactions, whereas previous sexually transmitted disease and new pregnancy were associated with decreased clearance.

**Conclusions:**

HPV16 was the most frequent genotype to cause an incident oral HPV-infection. Low risk HPV genotypes cleared from oral mucosa more quickly than high risk HPV genotypes. Pregnancy affected the outcome of oral HPV infection.

## Introduction

Human papillomavirus (HPV) infections are causally linked with head and neck cancers (HNSCC), particularly oropharyngeal cancers [1]–[3]. This implicated association necessitates natural history studies on oral HPV infection, because practically no longitudinal data exist on asymptomatic oral HPV infections. There are cross sectional studies on oral HPV prevalence but the results are quite divergent. We demonstrated in the early 1990′s that the detection rate of asymptomatic oral HPV was critically dependent on the sampling and HPV testing methods used, ranging from 3.8% to 29.4% [4]–[5]. Recently, Sanders and co-workers reported oral HPV prevalence of 7.3% in adult population in the US [6].

Oral HPV infections have been associated with the number of sexual partners, oral sex, deep kisses and hand warts [4] [7]–[8]. We were not able to find any association between oral sex and oral HPV infection among spouses [9].

The longitudinal Finnish Family HPV Study (FFHPVS) was designed to elucidate the dynamics of oral and genital HPV-infections within families [9]–[12]. Here we describe the key characteristics of genotype-specific incidence and clearance of oral HPV-infections as well as their predictors among the mothers followed for six years in FFHPVS. The present study is complementary to our recently report of the genotype-specific prevalence and persistence of the oral HPV-infection presented in the same cohort [13].

## Materials and Methods

### Women

**Figure 1:**
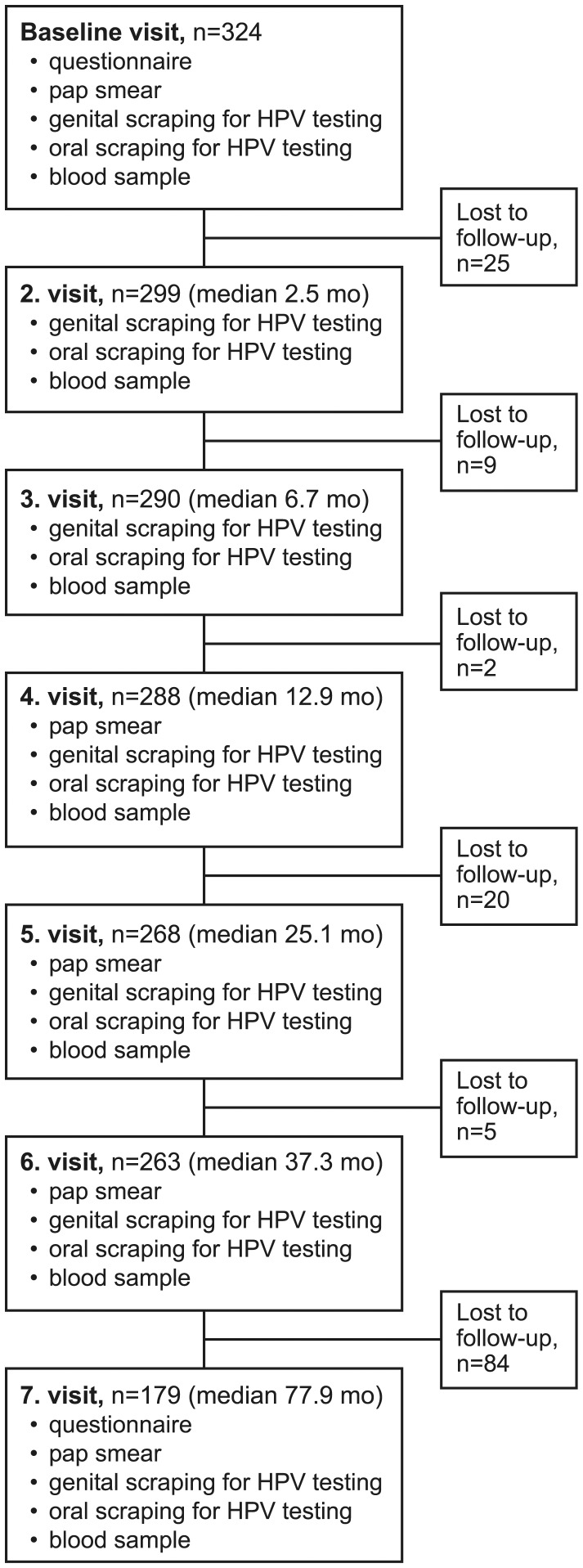
The flowchart of the study.

The FFHPVS is a longitudinal cohort study conducted at the University of Turku and Turku University Hospital, Finland previously described in detail [9]–[13]. In total, 329 families were enrolled, comprising 329 mothers, 131 fathers, and 331 newborns [9]. The inclusion criteria at recruitment were a minimum of 36 weeks of pregnancy and a written informed consent to participate in this study. Participants had to complete the minimum of two visits and women with only one visit were excluded. The Research Ethics Committee of Turku University Hospital approved the study protocol and its amendment (#2/1998 and #2/2006). The present analysis focused on oral HPV-infections of the mothers (mean age 25.5 years, median 26.0 years) during the 6-year follow-up (FU). The mean FU time for the cohort was 55.0 months. The flow chart of the study is shown in Figure 1. A structured questionnaire was introduced at baseline and repeated at 6-year visit (Table 1). The structured questionnaire included more than 60 questions recording the pertinent information on demographics, sexual behavior, gynecological and obstetric history as well as risk factors of HPV infections, to be tested as potential covariates in univariate and multivariate models. The spouses in this cohort were blinded by each other’s completed questionnaires. After entering the study, we did not exclude participants who separated from their spouse later. However, we recorded this information in the questionnaire introduced at the last follow-up visit and the data has recently been published as a separate study at 7 years (median 77.9 mo).

**Table 1:** Demographic data of the mothers.

CHARACTERISTICS	
Age at first visit after the delivery (n = 323)	25.5 yrs (median 26.0)
	Number (Percentage)
**Oral HPV positivity (any genotype)**	
Baseline (n = 324)	55 (17%)
2 mo follow-up (n = 299)	65 (21.7%)
6 mo follow-up (n = 290)	70 (24.1%)
12 mo follow-up (n = 288)	54 (18.8%)
24 mo follow-up (n = 268)	62 (23.1%)
36 mo follow-up (n = 263)	41 (15.6%)
72 mo follow-up (n = 179)	27 (15.1%)
**Marital status (n = 285)**	
Single	19 (6.7%)
Living with partner	132 (46.3%)
Married	131 (46.0%)
Divorced	3 (1.1%)
**Education (n = 285)**	
Compulsory school	24 (8.4%)
Vocational training	75 (26.3%)
Upper secondary school graduate	93 (32.6%)
College graduate	53 (18.6%)
Academic degree	40 (14.0%)
**Age at first sexual intercourse (n = 285)**	
<13 years	7 (2.5%)
14–16 years	160 (56.1%)
17–19 years	105 (36.8%)
>20 years	13 (4.6%)
**Number of lifetime sexual partners (n = 284)**	
0–2	70 (24.6%)
3–5	90 (31.7%)
6–10	65 (22.9%)
>10	59 (20.8%)
**Practices oral sex (n = 285)**	
Never	57 (20.0%)
Occasionally	193 (67.7%)
Regularly	35 (12.3%)
**Practices anal sex (n = 285)**	
Never	232 (81.4%)
Occasionally	51 (17.9%)
Regularly	2 (0.7%)
**Age at onset of oral contraception (n = 284)**	
Never	22 (7.7%)
<13 years	3 (1.1%)
14–16 years	117 (41.2%)
17–19 years	114 (40.1%)
>20 years	28 (9.9%)
**Smoking history (n = 284)**	
Never	142 (50%)
Current or past smoker	142 (50%)
**Use of alcohol (n = 284)**	
Never	28 (9.9%)
One dose per day	1 (0.4%)
One dose 2–3 times a week	28 (9.9%)
One dose per week	89 (27.5%)
One dose per month	138 (42.6%)
**History of sexually transmitted disease (STD) (n = 323)**	
No	261 (80.8%)
Yes	62 (19.2%)
**History of genital warts (n = 281)**	
No	201 (71.5%)
Yes	80 (28.5%)
**History of oral warts (n = 278)**	
Never	270 (97.1%)
Yes, no treatment	7 (2.5%)
Yes, surgical treatment	1 (0.3%)
**Skin warts (n = 164)**	
Hands	61 (37.2%)
Feet	64 (39.0%)
Multiple sites	39 (23.8%)

### Oral and Blood Samples

Oral scrapings were taken from the buccal mucosa of both cheeks and from the upper and lower vestibular area using a small brush (Cytobrush®, MedScan, Malmö, Sweden). The oral sampling was performed mainly by a research nurse. At visit 6 oral sampling was performed by a gynecologist and at visit 7 by two dentists (JW, LW). All who collected the oral samples were trained by the principal investigator of the study who is a dentist specialized in oral pathology. The brush was immersed in 80% ethanol, frozen and stored at -70°C [9]. Cervical and blood samples were collected in parallel with the oral samples as shown in flowchart of the study (Figure 1). The results on the serological data including details of the techniques used to analyse serum samples have been published earlier [14]–[15].

### HPV Genotyping

HPV-DNA was extracted from the oral scrapings with the high salt method as described previously [16]. Originally HPV-testing for the presence of any HR-HPVs was performed using nested PCR with MY09/MY11 as external and GP05+/GP06+ as internal primers [17]. The PCR products were hybridized with a digoxigenin-labeled HR-HPV-oligoprobe cocktail (HPV-types 16, 18, 31, 33, 35, 39, 45, 51, 52, 54, 56 and 58) to determine whether the samples were HR-HPV-positive (+) or -negative (-) [18]. The rationale for this approach was that when this study was started in 1998, there was no large-scale HPV genotyping method available as well as a high number of all samples (9,000). Nested PCR was performed for all oral samples as the viral load/cell and the number of infected cells among uninfected cells was expected to be much lower than in cervical samples. As GP05+/GP06+ primers might result in false positive amplification, all PCR products were also run on gel, transferred to filter and hybridized with HPV oligo cocktail of HPV types 16, 18, 31, 33, 35, 39, 45, 51, 52, 54, 56, 58. The hybrids were visualized with chemiluminescence reaction which exposed the X-ray film on filter. Using this method, we also could identify samples which were contaminated either during the sampling or PCR based on their similar intensity in the film. Altogether, we identified 23 samples that were contaminated during the sampling in the hospital. These samples were excluded.

HPV genotyping was performed with a Multimetrix kit® (Multimetrix, Progen Biotechnik GmbH, Heidelberg, Germany). Multimetrix kit® detects 24 LR- and HR-HPV-genotypes as follows: LR-HPV6, 11, 42, 43, 44, and 70; and HR-HPV16, 18, 26, 31, 33, 35, 39, 45, 51, 52, 53, 56, 58, 59, 66, 68, 73 and 82. The previous nested PCR products to detect any high-risk HPVs were biotinylated by re-amplification with GP05+/bioGP06+-primers. The labeled hybrids were analyzed with a Luminex LX-100 analyzer (Bio-Plex 200 System, Bio-Rad Laboratories, Hercules, USA). The assay was performed in half of the volume given in the protocol in all steps except the final one. A median fluorescence intensity (MFI) of at least 100 beads was computed for each bead set in the sample. The cut-off value for each run and HPV-type was 1.5× background MFI (negative control)+5MFI [17]. With serology, antibodies to the major capsid protein L1 of HPV types 6, 11, 16, 18 and 45 were analysed by multiplex HPV serology based on glutathione S-transferase fusion-protein capture on fluorescent beads, as described previously [19]–[20].

If any sample was positive for HPV16, then the protocol was repeated from the original sample using nested PCR and a bead-based HPV16 genotyping assay [21]. This assay was performed to rule out possible contamination with HPV16 during the previous tests due to several amplifications and the frequency of HPV16 in different samples.

### Definitions of Incident HPV Infection and HPV Clearance

An incident HPV-infection was recorded when a woman who was HPV-negative at baseline acquired an incident HPV infection during the FU. The incident event was recorded only once and the type detected at that special occasion was fixed as the “incident type”, completely irrespective what the subsequent events might have been. Among all women, clearance was defined as an event (at any FU visit) when a previously HPV-positive test turned out to be negative and remained HPV-negative until the end of the FU. Fluctuation was a pattern of HPV outcome in which consecutive samples were intermittently HPV+ and HPV- with different HPV genotypes, without any two consecutive samples being positive for the same or different viral genotype. Women with fluctuation were excluded in the statistical analysis.

### Statistical Analysis

All statistical analyses were performed using SPSS^®^ (SPSS, Inc., Chicago, USA, version 18.0.1) and STATA (Stata Corp., College Station, TX, USA, version SE11.0) software. Frequency tables were analyzed using the χ^2^-test with the likelihood ratio or Fisher’s exact test for categorical variables. Differences in the means of continuous variables were analyzed using non-parametric (Mann-Whitney or Kruskal-Wallis) tests for two and multiple independent samples, respectively.

Times (months) to incident and clearance events as well as genotype-specific incidence (IR) and clearance rates (CR) were calculated as recently described, expressed as events/1000 women months at risk (wmr) [12] [22]. To compare the individual IRs and CRs, the rate ratio (RR) statistic was used with test-specific 95% confidence intervals (95% CI).

We analyzed covariates of incident infections and virus clearance for species 7 and species 9 genotypes only, using population-averaged (PA) Poisson regression models for panel data, clustered by mother-ID, FU visit as the time variable, independent within-group correlation structure as the covariance pattern, and robust variance estimator (vce) to account for the within-subject correlation [23]–[24]. In the univariate model, we first tested all covariates recorded at baseline and selected variables from the FU questionnaire as potential risk factors of HPV [9]. In the final multivariate model, only significant univariates were entered, adjusted for age at study entry. All statistical tests were two-sided and considered significant at a *p*-value <0.05.

## Results

### Incident Infections

**Figure 2:**
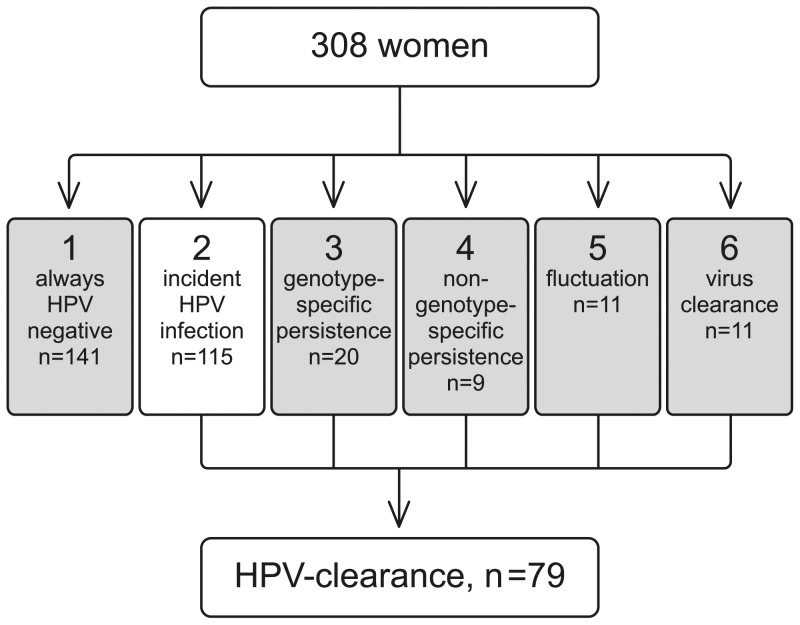
Outcomes of the oral HPV infections (n = 308, samples/participants with sufficient data for the analysis) in those 115 women (category 2), who developed an incident HPV-infection during the FU. The type-specific clearance focused only on the women who tested HPV-positive at least once during the FU and thus at risk for HPV clearance (category 2–6). Incidence, clearance and fluctuation were defined as explained in “Materials and Methods”. Genotype-specific persistence denotes any case with two (or more) consecutive FU samples positive for the same individual genotype as a single infection or as a part of a multiple-type infection, and non-genotype-specific persistence of consecutive FU samples positive for different genotypes, accordingly (results in details [13]).

**Figure 3:**
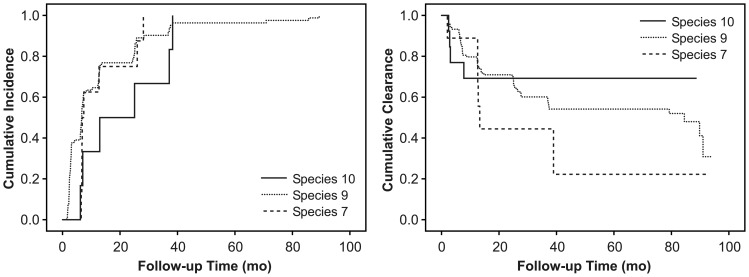
Cumulative incidence and clearance of oral HPV infections with species 7, 9 and 10 in Kaplan-Meier analysis.

Totally 171 mothers tested HPV-positive at some time point of the seven visits during the six-year FU. The mean FU time of the 115 women with incident infections (HPV negative at baseline) was 61.7±24.1 (SD) months (median 68.1, range 6.9–93.8). Outcomes of the study are presented in Figure 2. Incident infections of nine single HPV genotypes (HPV6, 11, 16, 18, 33, 56, 58, 66, 70) were detected. HPV16 was the most frequent, followed by multiple-type infections, in which HPV16 was involved in 57.1% (n = 8/14). HPV18 was less frequent than HPV16, but the time to incident events was nearly the same for both and was longer than for other HR-types (Table 2). Of the LR-HPV-types, HPV6 was the most common and had the longest mean time to first incident event (mean 11.4 months). Species 9 was the most dominant in incident infections, followed in frequency by species 7 and 10 (Fig. 3). The mean incidence time for species 7 and 9 was identical and was shorter than for species 10 (21.2 months).

**Table 2:** Times to incidence and clearance of oral HPV infections and their incidence rates for different HPV genotypes and species.

INCIDENT							Incident rate (IR) per 1,000 women months at risk (WMR)**			
	Incident infections		Mean time to 1st incident event (mo)*				Actuarial^3^ IR (95%CI)		Crude^4^ IR (95%CI)	
HPV genotype	N/Total	%	Actuarial^1^ (96%CI)		Crude^2^ (95%CI)		WMR	IR	WMR	IR
HPV6	5	4.3	23.9	11.4–36.5	23.9	6.2–41.7	8591	0.58 (0.07–1.11)	119.6	41.7 (5.9–77.4)
HPV11	1	0.9	7.0	7.0–7.0	7.0	7.0–7.0	8591	0.11 (0.01–0.34)	7.0	142.8 (111–402)
HPV16	75	65.2	13.3	9.3–17.3	13.3	9.2–17.4	8591	8.7 (6.7–10.7)	997.8	75.2 (58.7–91.5)
HPV18	5	4.3	14.8	4.9–24.7	14.8	0.9–28.7	8591	0.58 (0.07–1.11)	74.1	67.4 (10.4–124.7)
HPV31										
HPV33	2	1.7	6.6	5.8–7.4	6.6	1.2–11.9	8591	0.23 (0.08–0.55)	13.2	151.5 (42.2–349)
HPV35										
HPV44										
HPV45										
HPV51										
HPV52										
HPV56	2	1.7	7.5	0.0–18.0	7.5	2.1–12.9	8591	0.23 (0.08–0.55)	15.0	133.3 (38.6–305)
HPV58	6	5.2	11.1	7.6–14.6	11.1	6.6–15.6	8591	0.69 (0.02–1.26)	66.7	89.9 (21.1–157.9)
HPV59										
HPV66	2	1.7	13.3	0.0–35.4	13.3	2.1–24.6	8591	0.23 (0.08–0.55)	26.7	74.9 (24.7–172.8)
HPV70	3	2.6	8.9	5.3–12.6	8.9	0.9–17.0	8591	0.34 (0.04–0.74)	27.0	111.1 (74.2–229)
HPV73										
HPV82										
Multiple types	14	12.2	17.1	11.8–22.4	17.1	11.1–22.9	8591	1.63 (0.77–2.48)	239.1	58.5 (28.8–88.3)
**Total Incident Cases**	115	100.0								
**HPV Species:**										
Species 10 (6,11,13,44,55,74)	6	5.9	21.1	9.5–32.7	21.1	5.9–36.4	8591	0.69 (0.02–1.26)	126.7	47.4 (10.3–84.1)
Species 9 (16,31,33,35,52,58,67)	83	82.2	12.9	9.3–16.6	12.9	9.2–16.7	8591	9.66 (7.5–11.7)	1077.6	77.0 (61.0–92.9)
Species 7 (18,39,45,59,68,70,85)	8	7.9	12.9	6.3–18.9	12.6	4.9–20.3	8591	0.93 (0.28–1.57)	101.1	79.1 (26.5–131.8)
Species 6 (30,53,56,66)	4	4.0	10.4	0.0–20.9	10.4	6.6–27.4	8591	0.46 (0.19–0.92)	41.7	95.2 (64.6–184.0)
Species 5 (26,51,69,82)										
Species 11 (34,73)										
Total Incident Species	101	100.0					*RR		**RR	
*Comparison between Species using Rate Ratio (RR) test: Sp9 vs. Sp7: RR = 10.3 (5.74–18.58) p = 0.0001; Sp9 vs. Sp10:RR = 13.83 (7.36–25.99) p = 0.0001;Sp9 vs. Sp6:RR = 20.75 (10.28–41.85) p = 0.0001										
** Comparison between Species: Sp7 vs. Sp9 : RR = 1.02 (0.49–2.12) p = 0.900; Sp10 vs. p9: RR = 0.61 (0.27–1.39) p = 0.242; Sp6 vs. Sp9: RR = 1.23 (0.45–3.36) p = 0.640										
**CLEARANCE**							**Clearance rate (CR) per 1,000 women months at risk (WMR)**			
	**Cleared Infections**		**Mean Time to 1^st^ Clearance Event (Mo)**				**Actuarial** 3 **CR (95%CI)**		**Crude** 4 **CR (95%CI)**	
**HPV Genotype**	**N/Total**	**%**	**Actuarial** 3 **(95%CI)**		**Crude** 4 **(95%CI)**		**WMR**	**CR**	**WMR**	**CR**
HPV6	3/12	25.0	40.2	19.5–60.9	4.6	1.4–7.7	6647	0.45(0.05–0.96)	13.3	225.6(17.4–459)
HPV11	1/2	50.0	37.6	0–106.5	2.5	2.5–2.5	6647	0.15(0.01–0.44)	2.5	400.0(200–866)
HPV16	47/110	42.7	42.3	36.5–48.1	20.7	14.2–27.3	6647	7.01(5.05–9.08)	976.7	48.1(29.6–116.4)
HPV18	4/6	66.7	23.7	10.6–36.8	16.7	1.3–32.1	6647	0.69(1.2–1.19)	66.9	59.8(29.6–116.4)
HPV31										
HPV33	1/2	50.0	24.8	0.5–49.2	12.4	12.4–12.4	6647	0.15(0.01–0.44)	12.4	80.6(73.0–239
HPV35										
HPV42										
HPV43										
HPV44										
HPV45										
HPV51										
HPV52										
HPV56	1/3	33.3	38.9	35.6–42.3	37.2	37.2–37.2	6647	0.15(0.01–0.44)	37.2	26.9(25.2–79.2)
HPV58	8/9	88.9	26.0	9.7–42.4	18.8	9.8–27.8	6647	1.2(0.37–2.03)	150.4	53.3(17.4–89.2)
HPV59										
HPV66	3/5	60.0	29.0	0–65.2	16.8	0–35.6	6647	0.45(0.05–0.96)	50.4	59.5(5.8–125.8)
HPV70	2/3	62.5	39.2	0–91.3	12.7	12.4–12.9	6647	0.30(0.01–0.71)	25.3	79.0(26.3–186)
HPV73										
HPV82										
Multiple types	9/19	47.4	41.3	27.7–55.0	23.5	15.3–23.9	6647	1.35(0.47–2.23)	211.7	42.5(15.3–69.6)
Total	79/171	46.2	K–M, log rank: p = 0.572^a^		K–M, log–rank: **p = 0.032**					
	Fisher’s exact test: p = 0.158									
**HPV Species**	N/Total									
Species 10 (6,11,13,44,55,74)	4/14	28.6	39.8	20.7–58.9	4.0	1.4–6.5	6647	0.60(0.12–1.19)	16.1	248.4(37.8–462)
Species 9 (16,31,33,35,52,58,67)	56/121	46.3	40.8	35.3–46.3	20.3	14.7–25.9	6647	8.42(6.22–10.62)	1139.5	49.1(36.6–61.7)
Species 7 (18,39,45,59,68,70,85)	6/9	66.7	28.9	10.9–46.9	15.4	5.5–25.2	6647	0.90(0.18–1.62)	92.2	65.1(14.7–115.6)
Species 1(32,42)										
Species 8(7,40,43,91)										
Species 6(30,53,56,66)	4/8	50.0	31.8	10–53.8	21.9	5.2–38.6	6647	0.60(0.12–1.19)		45.6(19.3–88.9)
Species 5(26,51,69,82)										
Species 11(34,73)										
**Total**	70/152	46.1	K–M log–rank: p = 0.944		K–M, log–rank: **p = 0.015**		**RR		***RR	
	Fisher’s exact test: p00.353									
**Comparison between species using Rate Ratio (RR): Sp9 vs Sp7:RR:9.33(4.68–18.59) p = 0.0001; Sp9 vs Sp10: RR = 14.00 (6.47–30.25) p = 0.0001; Sp9 vs Sp6: RR = 14.00 (6.47–30.25)										
***Comparison between species: Sp7 vs Sp9: RR = 1.32 (0.57–3.07) p = 0.494; Sp10 vs Sp9: RR = 5.08 (2.04–12.65) p = 0.011; Sp6 vs Sp9: RR = 0.92 (0.33–2.55) p = 0.936										

* Time (mo) to the first incident event by HPV genotype among baseline HPV-negative women:

1 all baseline HPV-negative women at risk for incident event;

2 only women who developed an incident event;

** Incidence events per 1000WMR at risk;

3 all baseline HPV-negative women at risk,

4 only women who developed an incident event; the months at risk calculated i) until the first incident event (for those who had one), or ii) as total FU months for those with no incident event (Total WMR = 8591); IR, incidence rate; RR, Risk Ratio.

3 all women at risk for clearance of their HPV infection; months at risk being calculated 1)until the first clearance event, or 2) as total FU months for those with no clearance event (Total WMR = 6647);

4 only women with clearance event (months at risk calculated until the first clearance event); RR = Risk Ratio; including cases with no clearance (in Kaplan-Meier analysis total n = 171 and n = 152 for types and species, respectively); NC = non computable.

The highest IR was ascribed to HPV33, followed by HPV11, HPV56 and HPV70. The crude IRs of HPV16 and HPV18 were lower than for the other HR-HPV types. Due to this wide variation among individual types in different species, the crude IRs between the HPV species showed much less variation (Table 2).

The predictors of genotype-specific incident infections for species 7/9 are shown in Table 3. When all significant and borderline significant variables were entered in the multivariate Poisson PA model (including age), two variables retained their significance as independent protective factors of incident infections: 1) new pregnancy during FU and 2) having the same sexual partner during FU.

**Table 3:** Predictors of species 7- and 9-specific incident* oral HPV-infections in panel Poisson regression^1^ run in univariate mode and as adjusted for significant covariates.

Covariates	Incident Species 7 & 9 HPV Infections					
	CrudeIRR	95%CI	P	@AdjustedIRR	95%CI	P
Age (at study entry)	0.98	0.96–1.01	0.100	0.99	0.99–1.01	0.226
Mother seroconverted to HR-HPV (yes ref)	1.01	0.99–1.01	0.331			
Mother seroconverted to LR-HPV (yes ref)	1.01	0.99–1.01	0.327			
Mother seropositive to HR-HPV at baseline (yes ref)	0.85	0.44–1.49	0.510			
Mother seropositive to LR-HPV at baseline (yes ref)	0.71	0.35–1.41	0.336			
Baseline genital HR-HPV DNA status (+:ve ref)	1.01	0.50–2.03	0.979			
Baseline PAP smear (<ASCUS ref)	1.27	0.34–4.70	0.719			
Marital status at baseline (single ref)	0.99	0.99–1.01	0.327			
Employment status (employed; ref)	1.01	0.99–1.01	0.324			
Age at onset of sexual activity (<13 yrs ref)	0.99	0.99–1.01	0.336			
No. of sexual partners until 20 yrs old (0–2 ref)	1.01	0.99–1.01	0.325			
Life-time number of sexual partners	1.01	0.99–1.01	0.325			
Frequency of weekly intercourse (no trend)	0.99	0.99–1.01	0.357			
No. of deliveries in all partnerships	1.01	0.99–1.01	0.743			
Practices of oral sex (yes; ref)	1.01	0.99–1.01	0.421			
Practices of anal sex (regular; ref)	0.99	0.99–1.01	0.325			
Initiation of OC usage (<13 yrs ref)	0.99	0.99–1.01	0.345			
OC use (Y/N) (never ref)	0.99	0.99–1.01	0.323			
Smoking habits (never ref)	1.01	0.99–1.01	0.325			
Initiation of smoking (10–13 yrs ref)	0.99	0.99–1.01	0.388			
Consumption of alcohol (no ref)	0.99	0.99–1.01	0.323			
History of STD (yes ref)	0.99	0.99–1.01	0.323			
History of genital warts (yes; ref)	0.99	0.99–1.01	0.324			
History of oral warts (no history; ref)	1.01	0.99–1.01	0.323			
Second pregnancy during FU visit (no ref)	**0.13**	**0.03–0.55**	**0.005**	**0.23**	**0.05–0.96**	**0.045**
Change in marital status during FU	0.99	0.99–1.01	0.329			
Same sexual partner during FU (yes ref)	1.01	0.98–1.03	0.342	**1.04**	**1.01–1.06**	**0.0001**

Species 7 HPV genotypes: 18,39,45,59,68,70, and 85; Species 9 HPV genotypes: 16,31,33,35,52,58, and 67;

* Count outcome (incident event), as defined by the first incident event during FU.

1 Equivalent to GEE model using Poisson log-link for count outcomes, clustered by woman ID number, 95% CI calculated by robust estimation; @adjusted for age and all significant (and *borderline*) univariates in the model; IRR = incidence rate ratio.

### Clearance

The mean FU time for all infections was 59.1 months. Totally, 46.2% (n = 79) of the 171 mothers who tested HPV-positive at some visit during the FU, cleared their infection (Fig. 2). The mean FU time of these women was 60.0±24.4 (SD) months (median 65.5, range 6.7–92.0). By the end of FU, HPV58 had the highest clearance frequency (88.9%), whereas other HR-types had a clearance frequency above 33.3% (Table 2). The mean clearance time varied between the HR-types and was longest for HPV56. HPV6 had the lowest clearance frequency (25%) but a very short (4.6 months) clearance time. Only one of the HPV11-positive women cleared her oral infection (at 2.5 months). Nearly half of the multiple infections cleared within a mean of 23.5 months, which is similar to clearance time of HPV16 (20.7 months). Of the individual HPV species, the most prolonged crude clearance times were recorded for species 6 and 9, whereas species 10 had the shortest clearance time (4.0 months). Species 10 (LR-type) showed a significantly shorter but incomplete clearance as compared with species 7 and 9 (Figure 3). Due to the dominant role of HPV6 and 11, the crude CRs were highest for species 10 (Table 2).

Five variables were significant predictors of species 7/9 clearance: 1) older age (more common with increasing age), 2) history of atopic reactions (increased clearance), 3) history of STDs (decreased clearance), 4) frequency of weekly intercourse, and 5) new pregnancy (protective) during FU (Table 4). In the multivariate Poisson adjusted for age, all other variables except for the frequency of weekly intercourse retained their significance as independent predictors of species 7/9 clearance.

**Table 4:** Predictors of species 7 and 9-specific clearance* of oral HPV infections in panel Poisson regression^1^ run in univariate mode and as adjusted for significant covariates.

Covariates	Clearance of Species 7 & 9 HPV Infections					
	CrudeIRR	95%CI	P	@AdjustedIRR	95%CI	P
Age (at study entry)(young ref)	**1.06**	**1.01–1.16**	**0.004**	**1.05**	**1.01–1.10**	**0.009**
Mother seroconverted to HR-HPV (yes ref)	0.77	0.53–1.12	0.175			
Mother seroconverted to LR-HPV (yes ref)	1.35	0.86–2.11	0.179			
Mother seropositive to HR-HPV at baseline (yes ref)	0.66	0.35–1.66	0.216			
Mother seropositive to LR-HPV at baseline (yes ref)	0.95	0.49–1.83	0.892			
Baseline genital HR-HPV DNA status (+:ve ref)	1.18	0.47–2.96	0.713			
Baseline PAP smear (<ASCUS ref)	0.81	0.30–2.17	0.677			
Marital status at baseline (single ref)	0.96	0.72–1.26	0.773			
Employment status (employed; ref)	1.07	0.91–1.25	0.393			
History of atopic reactions (no**:** ref)	**1.59**	**1.09–2.30**	**0.015**	**1.71**	**1.17–2.51**	**0.005**
Age at onset of sexual activity (<13 yrs ref)	1.04	0.77–1.40	0.768			
No. of sexual partners until 20 yrs old (0–2 ref)	0.92	0.74–1.14	0.486			
Lifetime number of sexual partners	1.09	0.92–1.29	0.278			
Amount of weekly intercourse (0–1: ref)	**0.71**	**0.54–0.94**	**0.017**	0.78	0.58–1.05	0.111
No. of deliveries in all partnerships	1.02	0.75–1.39	0.851			
Practices of oral sex (yes; ref)	1.01	0.78–1.55	0.582			
Practices of anal sex (regular; ref)	1.01	0.68–1.52	0.923			
Initiation of OC usage (<13 yrs ref)	0.81	0.58–1.21	0.207			
OC use (Y/N) (never ref)	0.97	0.50–1.86	0.933			
Smoking habits (never ref)	0.99	0.68–1.44	0.985			
Initiation of smoking (10–13 yrs ref)	0.74	0.41–1.32	0.313			
Consumption of alcohol (no ref)	0.89	0.51–1.53	0.684			
History of STD (yes ref)	**0.69**	**0.47–0.99**	**0.047**	**0.63**	**0.42–0.95**	**0.031**
History of genital warts (yes; ref)	1.19	0.73–1.70	0.596			
History of oral warts (no history; ref)	1.13	0.66–1.92	0.652			
Second pregnancy during FU visit (no: ref)	**0.15**	**0.03–0.66**	**0.012**	**0.16**	**0.03–0.74**	**0.019**
Change in marital status during FU	*0.84*	*0.70–1.02*	*0.090*	0.95	0.77–1.17	0.669
Same sexual partner during FU (no ref)	0.98	0.54–1.77	0.952			

Species 7 HPV genotypes: 18,39,45,59,68,70,85; Species 9 HPV genotypes: 16,31,33,35,52,58,67;

* Count outcome (clearance event), as defined by the first clearance event during FU.

1 Equivalent to GEE model using Poisson log-link for count outcomes, clustered by woman ID number, 95% CI calculated by robust estimation; @adjusted for age and all significant (and *borderline*) univariates in the model; IRR = incidence rate ratio.

## Discussion

To our knowledge, there are no previous longitudinal studies on oral HPV-infections at the genotype level which hinder the comparison of our natural history data with that of others. As the six-year natural history of the cervical HPV-infections in these women is known [12] we are able to discuss the similarities and differences in viral outcomes at these two mucosal sites.

Altogether, 115/308 mothers developed an incident oral HPV-infection during FU (all outcomes in Figure 2). Considering the limited cross-sectional data [4] [8] [25]–[28], this was quite expected as there were seven oral samples/woman to be tested during FU. HPV16 is the most prevalent HPV-genotype in genital infections [29]–[30], also shown with the women in this study population [22], and not surprisingly, also among incident oral HPV-infections. HPV16 is also the most prevalent genotype in oral cancers and its proportion among head and neck cancers is higher than that among cervical cancers [1] [31]. In our cohort, the IR for HPV16 (75.2/1000 wmr) was higher than previously reported among 63 HIV-negative (1.7/100 wmr) or 136 HIV-positive (3.3/100 wmr) women who were followed-up for only 6 months [32]. Because the majority of incident infections cleared between 12–24 months, it is obvious that IRs at 6 months would differ significantly from those seen after 6 years. Other potential sources of variation include differences in oral sampling, and sensitivities of the HPV detection techniques [1]–[2] [5].

As always, there are limitations to this study. The number of the cohort (n = 329) is relatively small and not everyone had all samples available as well as some women were dropped out during the long six-year FU. This is why HPV infections with certain genotypes, e.g. HPV11, might be rare. This cohort is also unique as all women at baseline were pregnant. Hormonal changes during pregnancy might affect HPV infection and its outcome. FFHPVS included also the fathers and the infant of the index pregnancy. Thus, our woman-cohort represented mothers mostly with stable relationships. This has influence on their life style and sexual behavior which also reflect to the incidence and clearance of HPV infection. Also, the FU time of the study represents only a certain period of life of these women as all other studies as well. Thus the women’s HPV status before entering into the study is unknown as is the HPV status after the last six-year control visit. Furthermore, different body-sites (e.g. oral vs. genital/anal sites) may have an impact on HPV status at the other sites, and further analysis of the FFHPVS data is warranted for this in the future”.

In healthy, asymptomatic oral mucosa, the two most common non-invasive sampling methods for HPV testing are rinsing or brushing the mucosa. The rinsing of oral mucosa provides a sample of the exfoliated epithelial cells of which only part can be infected with HPV. Also the origin of the HPV infection cannot be determined as the detached cells can originate from any site of oral cavity and oropharynx. In the present study we are specifically interested in oral HPV infection. At least 100,000 cells are required for a representative sample and this may be difficult to accomplish with rinsing/gargle samples [4]–[5]. Rinsing and gargle samples are also heavily loaded with microbes as one gram of saliva might contain up to 10 million microbes which amount multiplies quickly when not correctly stored. Rinsing also results in a large range of cell amount among different samples. Furthermore, the detection of asymptomatic HPV infections requires infected basal or parabasal cells, which are difficult to acquire by rinsing and sometimes also with the means of brushing. Healthy oral mucosa consists of both keratinized and non-keratinized epithelia. Keratinized epithelium is more resistant to the collection of basal epithelial cells or even any nucleated cells and may produce false-negative results. Moreover, smokers’ oral mucosa might be more keratinized than that of nonsmokers. It is essential to brush non-keratinized surfaces, such as the buccal mucosa, the vestibules, the floor of the mouth, the border of the tongue (until the oropharynx), under the surface of the tongue and the keratinized and non-keratinized gingiva. Gingival or mucosal inflammation may lead to decreased keratinization and more easily detaching cells. Thus oral diseases unrelated to HPV and smoking might affect the HPV prevalence by the bias of sampling. The only truly reliable sample for HPV testing would be a representative mucosal biopsy sample. However, asymptomatic HPV infection may not cause any clinically visible changes and thus brushing might be the best method to screen for oral HPV infection.

The overall clearance of oral HPV infection was nearly the same as found in their cervix (46% versus 52%). However, genotype specific differences in clearance were found; oral LR-HPV genotypes were less likely to clear than those in the cervix [12] [22]. Those oral LR-HPV infections that cleared, did so quickly; HPV6 in 4.6 and HPV11 in 2.5 months. In cervix, HPV6 and HPV11 cleared within 14.8 and 12.4 months, respectively [12].

Pregnancy plays a special role in this cohort and a substantial proportion became pregnant again during FU. We showed previously that the prevalence of any oral HR-HPV infection was lowest during pregnancy (at baseline-visit) and increased after delivery [9]. Here we confirmed that a second pregnancy during FU decreased the probability of both incident oral HPV infections and their clearance (Tables 2 and 3), as found also in cervical HPV infections [22]. In our nested case control study focused on the second pregnancy of these mothers, we suggested that women committed to the second child did not share many of the known lifestyle behavioral risk factors of HPV infection [33], which supports the notion of Tenti and co-workers [34] who concluded that pregnancy as a general expression of more conscious social and sexual behavior might be a protective against HPV infections. Despite this protective effect of ongoing pregnancy against incident oral HPV infections, there must be a more mechanistic explanation for the decreased probability of clearance during the pregnancy, e.g., hormonal changes and/or immunosuppression, for which the second pregnancy is just a surrogate. Furthermore, immunological changes during pregnancy might hinder HPV clearance from the body [35]–[37].

Oral sex and open-mouth kissing have been implicated in increasing the risk of oral HPV infections [4] [7]–[8]. In this study, having the same sexual partner and a stable relationship during FU decreased the likelihood for incident oral HPV infections. We also found that HPV clearance was more common with increasing age, similarly as found in cervical HPV infections [38]–[40], albeit not confirmed by all studies [41]. Of interest is the emergence of atopic reactions among the independent predictors of increased clearance of species 7 and 9 oral infections (Table 3). One earlier study has shown atopic eczema to protect against HPV infections [42], whereas some more recent data have revealed an association between eczema and cervical cancer [43]. It is plausible to speculate that sensitized immunological hyper-reactivity in atopic patients might also prompt clearance of oral HPV by some yet unknown immunological mechanisms.

Finally, women with a history of previous STDs were less likely to clear their oral HPV infections. The two feasible explanations could be that 1) these women are more promiscuous than the others or 2) there are other co-infections interfering with HPV infection. However, in this analysis, we did not explore this issue in any further detail.

To conclude, this is the first study to characterize the incidence and clearance outcomes of oral HPV infections at the genotype level in a longitudinal setting. The predictors for these outcomes are somewhat similar to cervical HPV infections, being related not only to sexual behavior but also to pregnancy. Further comparative studies are needed to disclose all of the similarities and differences in the natural history of oral and genital HPV infections.
